# Single‐Stage Double‐Level Ilizarov Reconstruction for Distal Congenital Femoral Deficiency in Early Childhood: A Case Report

**DOI:** 10.1155/cro/6120792

**Published:** 2026-06-22

**Authors:** Nashat Aldzhamal, Ahmed Hassan Kamal, Karim Nashat

**Affiliations:** ^1^ Department of Orthopaedics, King Fahad Hospital Hofuf, Al-Ahsa, Saudi Arabia; ^2^ Division of Orthopaedics, Department of Surgery, College of Medicine, King Faisal University, Al-Ahsa, Saudi Arabia, kfu.edu.sa; ^3^ College of Medicine, Alexandria University, Alexandria, Egypt, alexu.edu.eg

**Keywords:** congenital femoral deficiency, Ilizarov technique, limb lengthening

## Abstract

**Background:**

Congenital femoral deficiency (CFD) with primary distal involvement (Paley Type 4) is the rarest phenotype in a spectrum that affects roughly 1 in 40,000–100,000 live births. Such children present early with pronounced limb length discrepancy (LLD), genu valgum, and patellofemoral maltracking.

**Case Presentation:**

We describe a 4‐year‐old girl who exhibited an 8 cm right‐sided LLD, severe distal femoral valgus, and hypoplasia of the lateral femoral condyle. A four‐ring Ilizarov frame was applied, and two corticotomies were performed in a single sitting: (1) an oblique distal osteotomy to open‐wedge correct valgus while permitting 1 mm day distraction and (2) a midshaft transverse osteotomy lengthened at 1 mm day. After 5 months, 12 cm of regenerated bone restored mechanical alignment and equalized limb length. The patient achieved full, painless knee and hip motion and resumed unrestricted play 7 months after frame removal. No deep infection, neurovascular injury, knee contracture, or regenerate fracture occurred.

**Conclusion:**

The case illustrates that carefully planned, simultaneous deformity correction and lengthening can safely deliver length gains beyond those typically reported in single‐level procedures while obviating multiple staged surgeries.

## 1. Introduction

Congenital femoral deficiency (CFD) is a rare, congenital anomaly of the lower limb which is caused by incomplete development of the femur and its surrounding soft tissues. According to the literature, CFD occurs in approximately one out of 40,000 to one out of 50,000 live births [1–3]. CFD can be categorized into several types; however, it is a diverse group of abnormalities, which can range from minor femoral shortening to total absence of the femur and other lower limb structures. The degree of severity varies depending upon the amount of skeletal involvement, the level of skeletal involvement, the presence or absence of soft tissue abnormalities, and whether or not the adjacent joints are involved. Proximal focal femoral deficiency (PFFD) accounts for the largest number of all congenital femoral deficiencies and therefore is the most common form of CFD. On the other hand, Distal femoral focal deficiency (DFFD) is extremely rare and poorly understood due to the lack of scientific literature describing only individual cases or small groups of individuals with this condition [4–7].

DFFD is characterized by either an absent or hypoplastic distal portion of the femur, while the proximal femur and surrounding hip structure remain intact [4–7]. The majority of patients develop LLD as well as other orthopedic problems during the first few years after birth. These include a wide variety of conditions including progressive LLD, genu valgus, knee instability, patella tracking problems, limited knee flexion, and abnormal gait mechanics secondary to asymmetric growth patterns at the distal end of the femur. Hypoplasia of both distal femoral condyles is most commonly seen in association with genu varus deformity. Many patients will go on to develop ligamentous insufficiencies in addition to trochlear dysplasia and/or patellar instability that will further impact knee function and make reconstructive surgical options even more difficult. Due to the fact that children’s bones continue to grow until late adolescence, the orthopedic deformity and associated LLD typically worsen with time and create a multitude of physical disabilities and emotional difficulties for affected children and their families [8].

In an attempt to classify and help plan for the appropriate treatments of CFD, multiple classifications have been developed. Dr. Pappas has previously identified DFFD as a Type IX [3]. More recently, Dr. Paley has developed a classification system that includes DFFD under the umbrella of Type IV CFD which he defines as “preservation of hip joint with predominant distal femoral involvement” [9]. Taylor et al. [5] modified Dr. Aitken’s [10] original classification to specifically define DFFD morphology and defined three different subtypes based on the spatial relationship between the distal femoral epiphysis and the femoral shaft. In Type A deformities, the distal femoral epiphysis continues anatomically with the shaft, although there is some degree of shortening. Type B deformities show preservation of the distal femoral epiphysis without anatomic continuity with the shaft. Finally, Type C deformities are characterized by a completely absent distal femoral epiphysis and marked femoral deficiency. It is important to accurately classify CFD because the degree of distal femur deformity, integrity of the knee joint, and anticipated difference in leg length at skeletal maturity affect choices available for treatment and ultimately determine the patient’s outcome.

Previous management for severe forms of CFD consisted mainly of predicting what would be expected in terms of leg length differences at skeletal maturity [8]. Those with minimal expected LLD’s were usually treated by wearing a shoe lift or performing contralateral epiphyseal ablation. However, those who had significant expected leg length differences were generally subjected to ablative surgical procedures, including amputation or Van Nes rotationplasty, so that they could receive optimal prosthetic rehabilitation. Although traditional ablative surgeries provided durable long‐term results in terms of functionality, they left patients with considerable esthetic, psychological, and societal ramifications.

Prior to recent advancements in limb reconstruction techniques utilizing Ilizarov’s tension stress principles, reconstructive surgical treatment for severe CFD was greatly limited. Monorail external fixation has traditionally allowed surgeons to gradually correct angular, rotational, and translational deformities while enabling controlled distraction osteogenesis and soft tissue adaptation [11]. The mechanical forces generated during controlled mechanical distraction promote angiogenesis, chondrogenesis, and intramembranous bone formation and consequently allow for large amounts of limb lengthening while protecting neurovascular structures and maintaining joint mobility [11]. Alberty et al. suggest that controlled mechanical distraction may also stimulate physeal activity and joint remodeling and therefore improve morphological characteristics of the distal femur and patellofemoral alignment during growth [12].

Although new technologies have improved our ability to manage patients with severe forms of CFD, reconstruction of DFFD remains technically challenging due to the rare nature of this condition, variable nature of distal femoral anatomy, associated ligamentous insufficiency, and increased risk of complications during prolonged periods of lengthening [4–6, 13]. Published reports have mostly focused on staged corrective procedures or isolated deformity corrections. There is also a paucity of data related to double‐level femoral reconstructions performed at two levels simultaneously in very young children with DFFD. Lastly, there is little information about how much potential for remodeling exists for hypoplastic distal femoral condyles during distraction osteogenesis or how the immature physis behaves postoperatively.

This report describes the successful treatment of a 4‐year‐old female child diagnosed as having Paley Type IV DFFD through the utilization of single‐stage double‐level Ilizarov reconstruction to treat her bilateral distal femoral valgus deformities simultaneously while achieving significant gains in femoral length. What makes this case unique is that simultaneous double‐level reconstruction was utilized in a very young child for the first time. Additionally, we achieved substantial increases in femoral length, restored nearly normal mechanical alignment, demonstrated progressive remodeling of her hypoplastic lateral femoral condyle, improved patellofemoral tracking, and preserved limb function without requiring ablative procedures. Therefore, this case contributes to the limited body of research related to strategies for managing limb reconstruction in children with DFFD and demonstrates biological remodeling capabilities existing for immature distal femora undergoing distraction osteogenesis.

## 2. Case Presentation

### 2.1. The Patient Profile

She is a 4‐year‐old girl who had a shorter right leg identified at birth which worsened over time with regard to gait. There were no complications during pregnancy, delivery, and neonatal periods. Her twin sister is normal.

She has no known family history of skeletal dysplasia or teratogenic exposure.

### 2.2. Clinical Findings

**Figure 1:**
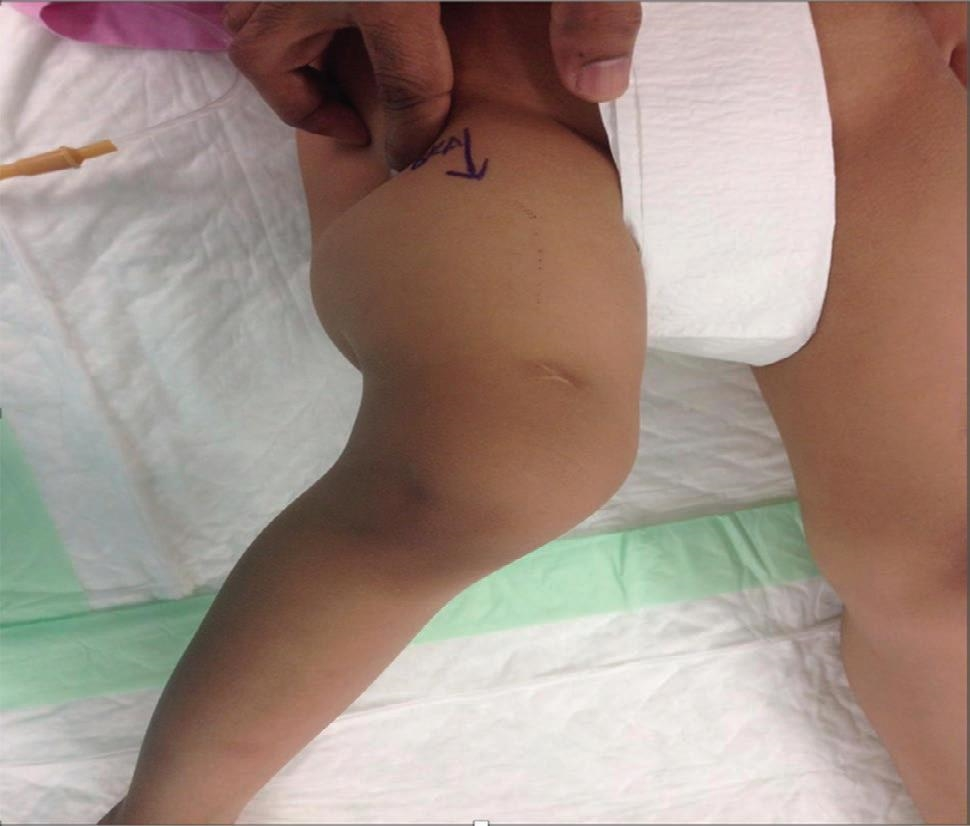
Preoperative photograph of the patient’s both lower limbs showing marked right valgus deformity.

Gait analysis by clinical observation revealed short limb gait with valgus collapse during the stance phase and reduced single limb support time. Right leg length was found to be short by 8 cm compared to the left (true LLD) (measured using standing full‐length radiographs). There is a valgus deformity of the right knee as seen in Figure 1. The mechanical axis (measured as the distance between the mechanical axis and the center of the knee joint) deviated 48 mm from the center of the knee joint, and there is approximately a 15° valgus mechanical axis angle. Symmetrical hip motions were present from 0° to 120° and pain‐free and stable. Fixed flexion deformity of the right knee occurred, and the patella tracked laterally. However, she still retains 30°–130° of knee flexion (range of motion measured using a standard goniometer) with severe medial laxity. Neurovascular exam of distal limbs was normal, and skin trophicity is normal.

### 2.3. Imaging

**Figure 2:**
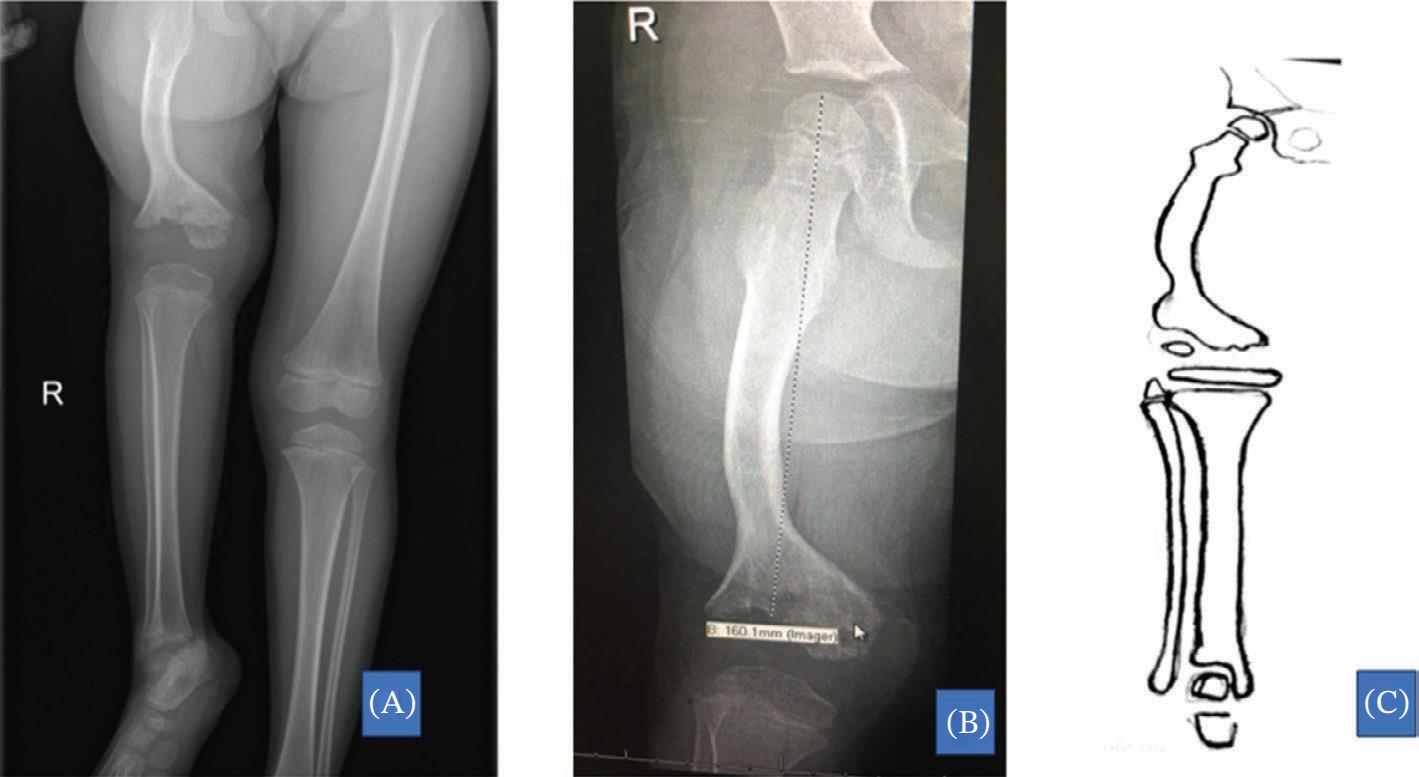
Preoperative (A) long‐leg AP radiograph and (B) femur and (C) drawing at the age of 4 years demonstrating overall right femoral shortening, hypoplasia of the lateral femoral condyle, shallow trochlea with patellar lateralization, and preserved proximal femoral anatomy.

Specifically, long‐leg radiographs identified (i) shortening of the entire length of the right femur, (ii) marked hypoplasia of the lateral femoral condyle, (iii) an underdeveloped trochlear groove that resulted in patella lateralization, and (iv) well‐developed proximal anatomy of the femur as well as coxa vara deformity **(**Figure 2A–C**)**. An MRI was performed to rule out physeal bar formation, and the results further supported the findings of hypoplastic lateral femoral condyles and confirmed that both cruciate ligaments were anatomically intact and oriented normally but were both underdeveloped, especially the ACL (Supporting Information Videos S1, S2, and S3). Routine blood tests (serum calcium, phosphate, alkaline phosphatase, thyroid function studies, and parathyroid hormone) were all within normal limits.

The following differential diagnoses were considered:

• PFFD
• Femoral hypoplasia–unusual facies syndrome
• Posttraumatic distal femoral physeal injury
• Congenital short femur syndrome
• Skeletal dysplasia associated with femoral shortening

## 3. Diagnosis and Classification

The diagnosis of DFFD was established based on the combination of progressive limb length discrepancy, valgus knee deformity, hypoplasia of the lateral femoral condyle, preserved proximal femoral anatomy, and MRI confirmation of an intact distal femoral physis without evidence of physeal bar formation. According to the Paley classification system, the preserved hip anatomy and predominant distal femoral involvement were consistent with Type IV CFD. Based on Taylor’s classification, the presence of a distal femoral epiphysis maintaining osseous continuity with the femoral shaft corresponded to Type A DFFD. Given the preserved hip function, intact proximal femoral anatomy, and distal femoral deficiency, the patient was considered an appropriate candidate for limb reconstruction. The projected limb length discrepancy at skeletal maturity, estimated using the multiplier method, was approximately 20–22 cm.

### 3.1. Surgical Technique

**Figure 3:**
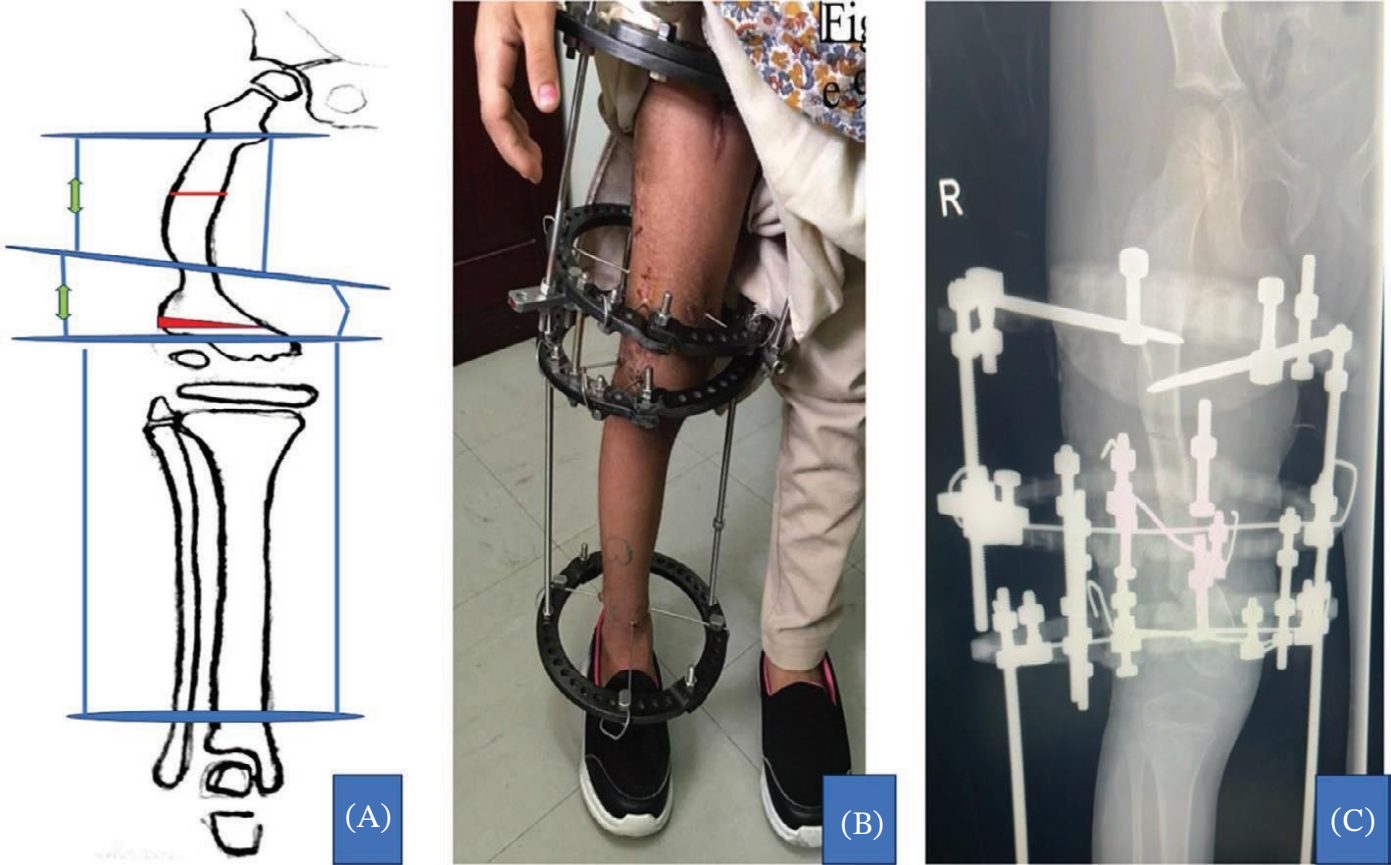
(A) Drawing and (B) photograph of Ilizarov frame assembly showing the four‐ring construct (three femoral and one tibial). Red lines indicate the cortectomy sites, and the green arrows indicate the distraction directions. (C) Immediate postoperative AP x‐ray.

After induction of general anesthesia, the limb was prepared and draped free to enable intraoperative testing of all movements. A four‐ring circular Ilizarov fixator was used. Three carbon fiber rings (each being 160 mm in diameter) were positioned distally to utilize as a form of distal fixation. The proximal area contained two “half” rings that formed an arch on top of the proximal femur for use as a form of proximal fixation. Tensioned wires (1.8 mm) were placed distally for a total of six wires with each wire under a tension of 110–130 kg. Two 5 mm half pins were utilized for proximal fixation and entered through safe anterolaterally located corridors (Figure 3A–C).

A 4 cm lateral incision was made 1 cm proximal to the distal growth plate to allow subperiosteal access to perform the low‐power oblique corticotomy. The oblique corticotomy was performed at approximately 45° relative to the femoral shaft axis from the lateral to the proximal side of the bone to the medial to the distal side of the bone. Intraoperatively, the 4 mm opening wedge was placed to correct the patient’s preoperative valgus deformity. Gradual distraction began on the third postoperative day by advancing the proximal fragment 1 mm per day in four increments.

**Figure 4:**
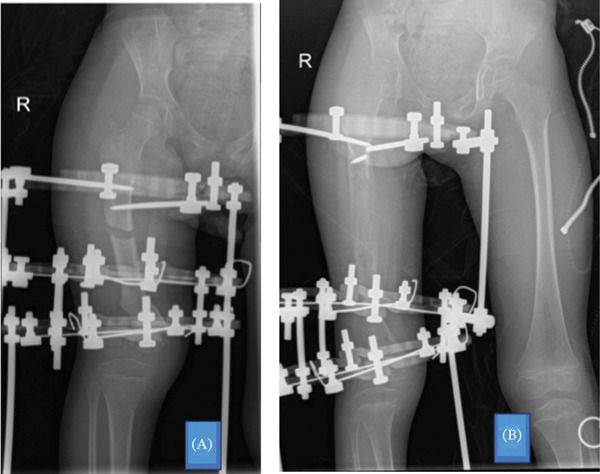
Serial AP radiographs during distraction at (A) 2 weeks and (B) 12 weeks showing gradual opening of the distal wedge and progressive lengthening at the midshaft level with visible regenerated bone formation.

The midshaft transverse corticotomy was performed through a 2 cm anterolaterally placed stab incision. The femoral midshaft diaphysis was drilled multiple times, and an osteotome was used to complete the osteotomy (Figure 4). On Day 3, a distraction of 1 mm/day (0.25 mm × 4) was initiated. Prophylactic antibiotics (cefazolin 25 mg/kg) were administered for 24 h.

Total operative time was 120 min with blood loss of less than 100 mL with a cumulative fluoroscopy exposure time of approximately 2.5 min. No intraoperative neurovascular, technical, or anesthetic complications occurred.

### 3.2. Postoperative Course and Follow‐Up

Ankle and hip continuous passive motion exercises commenced on Day 1 after surgery, and partial weight bearing was permitted by Week 7. Daily pin site cleaning with a 2% chlorhexidine solution was done by parents. Radiographic assessments every week were used to determine whether or not to make any changes to the distraction process (2 weeks [Figure 4A] and 12 weeks [Figure 4B**]**).

**Figure 5:**
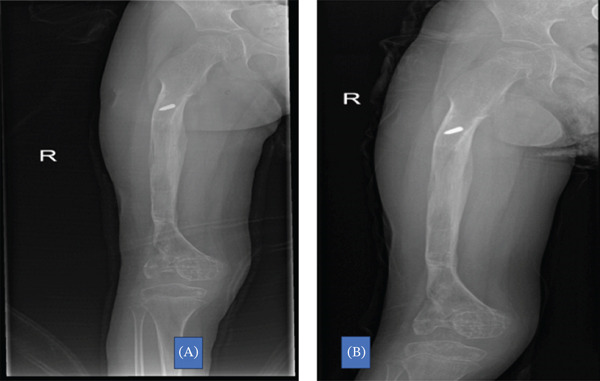
Radiograph (after frame removal) at (A) 7 months and (B) 8 months confirming solid regenerate consolidation across both corticotomy sites and restoration of femoral length; the knee joint line is horizontal and congruent.

**Figure 6:**
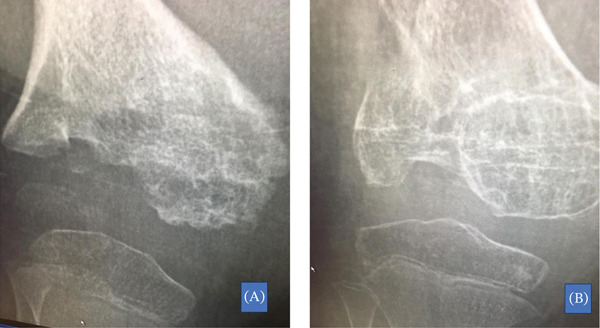
Twelve‐month follow‐up radiograph showing development of the lateral condyle and centralization of the (B) patella compared to the (A) preoperative x‐ray.

The distal wedge was able to centralize the mechanical axis to within 4 mm of the center of the knee by Week 12, and the lateral femoral condyle had significantly developed based upon the x‐ray; the patient was then allowed to resume full weight bearing while still in the frame. The cumulative length gained (length gain measured radiographically between corticotomy sites) from distraction by Week 22 was 12 cm (the distal segment was 6 cm, and the midshaft segment was 6 cm). The distraction index was 33 days per centimeter (distal) and 30 days per centimeter (midshaft). Consolidation to three cortices was obtained and allowed for frame removal during anesthesia at Month 7 (Figure 5A). Following frame removal, the patient wore a functional brace for an additional 6 weeks. Follow‐up x‐rays demonstrated continued consolidation of the regenerate (Figure 5B) and increased development of the lateral femoral condyle and further centralized position of the patella (Figure 6B) compared to preoperative x‐ray (Figure 6A).

**Figure 7:**
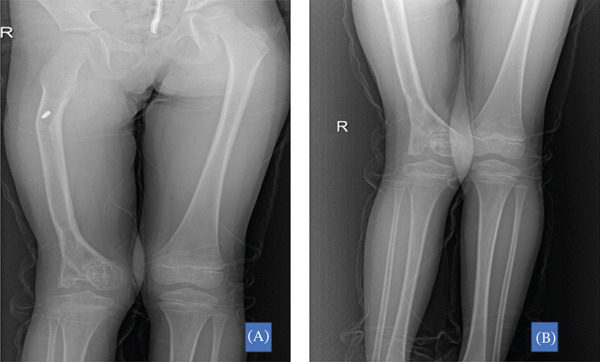
Eighteen‐month follow‐up radiograph showing alignment of (A) both lower limbs showing (B) both femurs showing both knees.

Complications were limited to two superficial pin site infections (Gladstone Grade 1) that resolved with oral cephalexin and breakage of one of the femoral Schanz pins. There was knee stiffness which was improved by physiotherapy. No joint subluxation, neurovascular deficit, premature consolidation, or late fracture was observed within 18 months’ follow‐up (Figure 7A,B). The child resumed normal kindergarten activities without gait aids with normal patellar tracking and full range of motion at the knee (Table 1) (Supporting Information Videos S4 and S5). Parents reported high satisfaction regarding limb appearance, gait improvement, and overall function.

**Table 1:** Sequence of radiological and functional outcomes.

Time point	LLD	MAD	Knee ROM	Weight bearing	Key findings
Preoperative	8 cm	48 mm lateral	30°–130°	Full	Valgus deformity, lateral patellar tracking
3 months	4 cm	10 mm lateral	20°–120°	Partial	Active distraction and regenerate formation
6 months	1 cm	4 mm lateral	10°–130°	Full	Condylar remodeling and improved tracking
12 months	< 1 cm	Near neutral	0°–135°	Full	Consolidated regenerate
18 months	< 1 cm	Neutral	0°–135°	Full	Independent gait and stable alignment

### 3.3. Patient Timeline

Detailed patient time is summarized in Table 2.

**Table 2:** Patient timeline.

Time point	Clinical event
Birth	Right lower limb shortening noted at birth
Infancy	Progressive limb length discrepancy and valgus deformity observed
Age 4 years	Referral for orthopedic evaluation
Preoperative assessment	Standing radiographs and MRI confirmed Paley Type IV DFFD with 8 cm LLD
Surgery	Single‐stage double‐level Ilizarov reconstruction performed
Postoperative Day 1	Physiotherapy and ROM exercises initiated
Postoperative Day 3	Gradual distraction initiated at 1 mm/day
Week 7	Partial weight bearing allowed
Week 12	Mechanical axis correction and condylar remodeling observed
Week 22	Completion of distraction phase with 12 cm total length gain
Month 7	Frame removal after radiographic consolidation
Month 8	Full weight bearing without frame
Month 12	Improved patellar tracking and condylar development confirmed radiographically
Month 18	Independent gait, full ROM, and return to unrestricted activities

## 4. Discussion

There are few publications related to this area, due to the relative rarity of DFFD; however, the majority of those available have been limited to either single case reports or small series of cases since the original work published by Tsou and Gilsanz in the early 1980s [7]. As such, many aspects of the natural history of DFFD, the best method of classification, and the most appropriate methods of reconstruction are poorly defined. While the majority of patients with DFFD exhibit similar clinical features such as hypoplasia of the distal femoral condyles, progressive limb length discrepancies, genu varum or valgum, patellofemoral instability, and preservation of the anatomy of the proximal femur and hip [4–7], the current case exhibited all of these classic features including significant hypoplasia of the lateral femoral condyle, genu valgum, a shallow trochlea, and lateral patella maltracking.

All of the imaging findings observed in the current case are consistent with previous DFFD cases. Taylor et al. stated that valgus deformity and dysfunction of the extensor mechanism often occur secondary to asymmetrical distal femoral growth and hypoplasia of the lateral femoral condyle [5]. Similarly, MRI in the current case found that the patient’s cruciates were intact, but there was ligamentous hypoplasia, and there was no evidence of a physeal bar. These findings have also been noted in other DFFD cases [4–7].

Preservation of physeal continuity was an important finding in the current case. Continued growth potential and response to physeal stimulation are critical components in successful limb reconstruction and remodeling after distraction osteogenesis.

Classification systems are critical to treatment planning and prognosis in patients with CFD. The current case was classified as Paley Type IV CD with preserved hip anatomy and predominantly distal femoral involvement [9]. Based upon Taylor’s modification of the Aitken classification system, this deformity is equivalent to Type A DFFD because even though the distal femoral epiphysis had a considerable amount of osseous continuity with the femoral shaft, it still underwent substantial shortening [5]. Additionally, the anticipated leg length difference at maturity was expected to exceed 20 cm. Historically, individuals with differences greater than 20 cm have been considered candidates for ablative treatments such as rotationplasty or amputation [8]; however, recent advancements in limb reconstruction utilizing Ilizarov principles have progressively broadened the indications for limb‐sparing surgical interventions in select patients who possess stable joints and adequate soft tissue function [9, 14, 15].

Distraction osteogenesis and physeal remodeling likely played significant roles in achieving the positive radiographic and functional outcomes observed in this case. Ilizarov has proposed that slow controlled mechanical distraction can induce angiogenesis, proliferation of mesenchymal stem cells, intramembranous bone formation, and regeneration of adaptive soft tissues [14]. Experimental studies have also shown that slow distraction can stimulate proliferation of physeal chondrocytes and promote remodeling of adjacent epiphyseal structures [12]. During the course of this case study, we were able to observe progressive maturation of the hypoplastic lateral femoral condyle and improved patellar tracking centrally. We believe that gradual mechanical deloading in combination with physeal stimulation may have induced adaptive remodeling of the immature distal femur. Furthermore, restoring more normal biomechanical alignment may have altered joint loading forces across the knee, thereby possibly contributing to improved patellofemoral tracking and condylar growth.

Several advantages existed for performing a single‐stage double‐level Ilizarov reconstruction in this patient. Correction of valgus deformity and reduction in limb length difference simultaneously decreased the overall treatment burden and eliminated the need for multistage procedures and subsequent anesthesia exposures. The utilization of two corticotomies created separate regenerate zones for each level, thereby potentially lessening mechanical stresses placed on a single distraction site as well as lessening the likelihood of regenerate zone failure. An oblique distal corticotomy provided simultaneous correction of angle and lengthening while preserving distal femoral biology and minimizing additional osteotomies. For very young children with open physis and large amounts of modeling potential, circular external fixation may offer a viable limb‐sparing option compared to ablative procedures.

As previously mentioned, this approach has potential risks. Complications commonly seen with lengthening in congenital deficiencies include pin site infections, joint stiffness/subluxation, neurovascular injuries, premature consolidation/regenerate zone insufficiency, fractures, and recurrence of deformity [9, 14, 15]. The current patient experienced mild complications consisting solely of superficial pin tract infections and transient knee stiffness which both resolved with nonoperative care. It is worth noting that despite preoperative ligamentous hypoplasia and knee instability, no postoperative knee subluxations or neurovascular complications arose. We attribute this good outcome to selective patient criteria, rigidly constructed frames around the knees, slow distraction rates, and closely monitored x‐rays throughout treatment.

While the results obtained in this case study are encouraging, several important caveats exist. This is a single case report without controls thereby limiting the generalizability of our data. Additionally, we acknowledge a bias towards selecting patients with better‐than‐average proximal anatomy, open physics, and satisfactory soft tissue function to allow for reconstruction thereby excluding some more severely affected forms of DFFD such as Taylor Type C deformities. Lastly, while we have followed this patient for an extended period (approximately 1.5 years), we do not feel confident in stating long‐term outcomes concerning physeal behavior, recurrence of valgus deformity, progression of limb length difference, and maintenance of joint congruence. Lastly, while we did see radiographic evidence for progressive remolding of the hypoplastic lateral femoral condyle, we could not utilize advanced quantitative imaging analyses or formal gait lab evaluations to assess these changes.

## 5. Conclusion

This case illustrates that DFFD, a rare form of CFD, can be successfully treated with limb‐preserving techniques in appropriately selected children. The patient’s substantial restoration of lower extremity length, correction of the valgus deformity, and improvement in knee function achieved through the use of circular external fixation demonstrate the potential for limb reconstruction in selected children with DFFD. Furthermore, the distal femoral and patellofemoral remodeling demonstrated in the patient’s radiographic studies are consistent with the growing body of evidence demonstrating that limb reconstruction alternatives may extend the range of treatment options for DFFD beyond traditional ablative solutions.

**Nomenclature**
ACL: anterior cruciate ligament
AP: anteroposterior (refers to x‐ray view)
CFD: congenital femoral deficiency
DFFD: distal femoral focal deficiency
LLD: limb length discrepancy
MRI: magnetic resonance imaging
PFFD: proximal focal femoral deficiency

## Funding

No funding was received for this manuscript.

## Disclosure

This case report has been prepared in accordance with the CARE guidelines.

## Ethics Statement

According to guidance received from the Institutional Review Board (IRB), Research and Studies Department, Al‐Ahsa Health Cluster, in January 2026, formal ethical approval was not required for this single‐patient case report.

## Consent

Written informed consent for participation and publication of the patient’s clinical information, radiographic images, and accompanying materials was obtained from the patient’s legal guardian.

## Conflicts of Interest

The authors declare no conflicts of interest.

## Supporting Information

Additional supporting information can be found online in the Supporting Information section.

## Supporting information


**Supporting Information 1.** Video S1: Coronal MRI T2 sequences confirming hypoplastic lateral condyle, shallow trochlea, and absence of physeal bar.

**Supporting Information 2.** Video S2: Coronal MRI T1 sequences confirming hypoplastic lateral condyle, shallow trochlea, and absence of physeal bar.

**Supporting Information 3.** Video S3: Sagittal MRI T1 sequences confirming hypoplastic lateral condyle, shallow trochlea, and absence of physeal bar.

**Supporting Information 4.** Video S4: Gait at 18 months following surgery.

**Supporting Information 5.** Video S5: Knee tracking at 18 months following surgery.

## Data Availability

The data that support the findings of this study are available from the corresponding author upon reasonable request.
